# Longitudinal sampling of external mucosae in farmed European seabass reveals the impact of water temperature on bacterial dynamics

**DOI:** 10.1038/s43705-021-00019-x

**Published:** 2021-06-21

**Authors:** Daniela Rosado, Raquel Xavier, Jo Cable, Ricardo Severino, Pedro Tarroso, Marcos Pérez-Losada

**Affiliations:** 1 CIBIO-InBIO, Centro de Investigação em Biodiversidade e Recursos Genéticos, Universidade do Porto, Campus Agrário de Vairão, Vairão, Portugal; 2 School of Biosciences, Cardiff University, Cardiff, UK; 3 Piscicultura Vale da Lama, Sapal do Vale da Lama, Odiáxere, Lagos, Portugal; 4 Computational Biology Institute, Department of Biostatistics and Bioinformatics, Milken Institute School of Public Health, George Washington University, Washington, DC USA

**Keywords:** Microbiome, Metagenomics

## Abstract

Fish microbiota are intrinsically linked to health and fitness, but they are highly variable and influenced by both biotic and abiotic factors. Water temperature particularly limits bacterial adhesion and growth, impacting microbial diversity and bacterial infections on the skin and gills. Aquaculture is heavily affected by infectious diseases, especially in warmer months, and industry practices often promote stress and microbial dysbiosis, leading to an increased abundance of potentially pathogenic bacteria. In this regard, fish mucosa health is extremely important because it provides a primary barrier against pathogens. We used 16 rRNA V4 metataxonomics to characterize the skin and gill microbiota of the European seabass, *Dicentrarchus labrax*, and the surrounding water over 12 months, assessing the impact of water temperature on microbial diversity and function. We show that the microbiota of external mucosae are highly dynamic with consistent longitudinal trends in taxon diversity. Several potentially pathogenic genera (*Aliivibrio, Photobacterium, Pseudomonas,* and *Vibrio*) were highly abundant, showing complex interactions with other bacterial genera, some of which with recognized probiotic activity, and were also significantly impacted by changes in temperature. The surrounding water temperature influenced fish microbial composition, structure and function over time (days and months). Additionally, dysbiosis was more frequent in warmer months and during transitions between cold/warm months. We also detected a strong seasonal effect in the fish microbiota, which is likely to result from the compound action of several unmeasured environmental factors (e.g., pH, nutrient availability) beyond temperature. Our results highlight the importance of performing longitudinal studies to assess the impact of environmental factors on fish microbiotas.

## Introduction

Cumulative evidence shows that the diversity of commensal microbiota mirror fish health and that microbial diversity can improve host fitness.^1–3^ Fish microbial composition and structure are driven by host related factors, including host taxonomy and ontogeny,^4,5^ but also physio-chemical properties of the water (e.g., temperature, pH, oxygen and nutrient concentrations^6–9^), which can be highly dynamic.^10^ Consequently, the microbiota of fish external mucosa, i.e., skin and gills, can be highly variable.^11^ Water temperature, in particular, can be pivotal as it can prompt modifications in the composition of key components of the fish skin, such as mucins and mucosal immunoglobulins, that influence the microbiota by limiting bacterial adhesion and growth.^12–15^ In addition, bacteria have optimal temperature ranges that maximize their growth,^16^ thereby conferring some taxa with a competitive advantage and creating colonization opportunities for other taxa during temperature shifts.^17^ Several microbiome studies have reported direct or indirect temperature-related changes in fish bacterial diversity of the skin, gill or gut in the puffer fish,^18^ brown trout,^19^ tilapia,^6^ yellowtail kingfish,^20^ chub mackerel,^9^ and other teleost species.^21^ Importantly, differences in host’s tolerance to temperature seem to be related to microbial composition and their resilience to temperature changes.^6^ Such studies were either based on experimental setups to test the effects of specific temperatures (22 and 26 °C;^20^ 12 and 24 °C;^6^ 10, 20, and 29 °C^18^) or on sampling single time points at different localities;^19,21^ the only exception being Minich et al.^9^ who conducted a year long study of the chub mackerel microbiota. Hence, the effects on fish microbiota of natural fluctuations in sea water temperature are still largely understudied, although there are increasing reports of a positive correlation between temperature and the abundance of potentially pathogenic genera (e.g., *Vibrio* and *Flavobacterium*^18^ and *Photobacterium*^9,20^). Indeed, several bacterial infections in fish have seasonal distributions with higher incidence in warmer months (e.g.,^22–24^). This pattern is particularly notable in farmed fish (e.g.,^25–28^), where the abundance of pathogenic taxa is higher compared to their wild host counterparts.^29^

Aquaculture is the fastest growing food-production industry; however, its sustainability is heavily affected by infectious diseases.^30^ Fish farming practices are known to promote microbial dysbiosis, which often involves an increase in the abundance of potentially pathogenic bacteria and a parallel decrease in microbial diversity (e.g.,^31^). This is critical in fish farms given the predicted increase of diseases due to the ongoing rise of sea surface temperatures.^32,33^ In this regard, fish mucosa health is of extreme importance, particularly in the case of the skin and gills, since both act as primary physical and chemical barriers against pathogens.^34^ Specifically, mucosal microbiota can have an important role in controlling pathogen abundances either through direct competition or antibiotic production.^35,36^ Indeed, putative bacterial pathogens will integrate within the microbiota of healthy/asymptomatic fish, where their abundance is controlled by harmless commensal bacteria (e.g.,^37,38^).

The European seabass (*Dicentrarchus labrax*) is one of the most profitable species farmed in the Mediterranean region, including southern Portugal.^39^ Being a eurythermic (from 5 to 28 °C) and euryhaline (from 3 to 35%) species, it is traditionally farmed in outdoor ponds located in protected areas, such as estuaries and coastal lagoons, or in open-water sea cages.^39^ As such, farmed fish are subjected to natural fluctuations of water physio-chemical properties. Among the many diseases reported in European seabass fish farms, the most frequent have bacterial etiological agents, with vibriosis (caused by *Vibrio* spp.) being the most prevalent, followed by photobacteriousis (*Photobacterium damselae*) and Tenacibaculosis (*Tenacibaculum maritimum*) (see^23,40^). Temperature and seasonality are considered the main epizootiological risk factors for severe disease caused by these pathogens.^23^

Here we used 16S rRNA high-throughput sequencing to characterize the composition and structure of the skin and gill microbiomes of the farmed European seabass and its surrounding waters over 12 months. We then assessed the impact of water temperature oscillations measured during different time frames (days, weeks, and months prior to sampling) on microbial diversity and function, as well as the dynamics and effects of potentially pathogenic bacterial genera in the microbial community.

## Materials and methods

### Experimental design, sampling, and processing

European seabass specimens were sampled between February 2017 and January 2018 from a single exterior pond of a fish farm operating in a semi-intensive regime and located in the Alvor Estuary (Portimão, Portugal). Due to the impossibility of sampling the same individuals throughout time, a random subset of five fish was sampled every fortnight, totaling 120 fish and 24 sampling time points. Fish were caught using a fishing rod and skin and gill mucous samples taken immediately using tubed sterile dry swabs (Medical Wire & Equipment, UK). Water samples (1 L) were concomitantly collected and filtered through 0.2 µm filters, except through December and once in February, due to technical constraints. Fish diet composition remained stable throughout sampling (SKRETTING/SORGAL), although pellet size increased from 4 to 6 mm. All sampled fish were considered healthy throughout the sampling period based on the lack of visible disease symptoms and mortality fraction recorded at the sampling pond (i.e., 105 fish, 29% of total density, died in May 2017 from unknown causes). Fish were not vaccinated against bacterial diseases and no antibiotics were administered during the sampling period. Water temperature (°C) was measured every 10 s in situ throughout the duration of the experiment using a probe placed in the pond at 20 cm deep. The mean daily temperatures ranged between 13.3 °C in January (the coldest month) and 25.1 °C in June (the warmest month), with an annual mean temperature of 18.6 ± 2.8 °C (Fig. S1).

Total DNA from 261 samples (120 skin, 120 gill, and 21 water filters) was extracted using the PowerSoil DNA Isolation Kit (QIAGEN, Netherlands). DNA extractions were shipped on dry ice to the University of Michigan Medical School (USA) for amplification of the V4 hyper-variable region of the 16S rRNA gene (~250 bp) according to the protocol of Kozich et al.^41^ All samples were sequenced in a single run on the Illumina MiSeq sequencing platform.

### Data processing and statistical analysis

Raw FASTQ files were denoised using the DADA2 pipeline in R vs. 4.0.2.^42^ Microbial composition (alpha-diversity) was calculated using Shannon and Faith’s phylogenetic diversity (PD) as implemented in the R package phyloseq.^43^ Microbial structure (beta-diversity) was estimated using phylogenetic Unifrac (unweighted and weighted) distances. Variations in microbial composition and microbial structure between sampling dates and months were assessed using the Kruskal–Wallis^44^ and PERMANOVA^45^ tests, as implemented in the adonis function of the vegan R package.^46^ Pairwise comparisons were done between all sampling dates and months using the Wilcox tests with Benjamini–Hochberg correction (stats package;^47^) and pairwise adonis.^48^ No significant differences were found in bacterial diversity between dates within months (results not shown). Additionally, dissimilarity in microbial structure between samples was visualized using Principal Coordinates Analysis (PCoA).

We used the software FEAST^49^ to estimate microbial monthly exchange between skin, gills and water. This software implements an expectation-maximization algorithm^50^ which infers the fraction of the microbiota (i.e., the sink) originating from different available sources (see Supplementary File 1 for more details).

Genera containing important bacterial pathogens affecting aquaculture were identified according to the list compiled by Haenen.^51^ Several of these potentially pathogenic (PP) genera showed peaks of abundance throughout the year: *Aliivibrio*, *Photobacterium, Pseudomonas,* and *Vibrio*. Temporal Insights into Microbial Ecology (TIME;^52^) was used to visualize the temporal dynamics and predicted microbial competition involving the most abundant PP genera (≥5%, hereon termed PP genera), as well as to assess their influence in the community structure throughout months (see Supplementary File 1 for more details).

The impact of temperature and season (coded as a categorical variable) on microbial alpha-diversity was assessed using linear models (*lm*), while their impact on microbial beta-diversity was assessed using permutational multivariate analysis of variance (PERMANOVA) (see Supplementary File 1 for more details). Several temperature variables were derived from the mean (mn) and standard deviation (SD) of the temperatures measured at different time frames preceding sampling dates. Briefly, we used temperatures from the 2 and 7 days before each sampling date (T2_mn/ T2_SD and T7_mn/T7_SD). We averaged diversity values per month since no significant differences within months were found and, consequently, decreased the impact of serial correlation in the analyzes. We assessed the influence of monthly temperature using samples grouped by month and temperature variables derived from the periods preceding each of the two sampling points in a given month (T4_mn/T4_SD and T14_mn/T14_SD). In addition, we tested the effects of monthly mean temperature and respective standard deviation (T_month_mn/T_month_SD) and of a broader categorical time-frame created by dividing the year into cold or warm months based on the daily annual average temperature (18.6 °C, see Supplementary File 1 for more details).

We used generalized least squares models (*gls*) from the nlme R package^53^ to estimate the relation between bacterial diversity and the abundance of PP genera with temperature. For this test, matrices containing pairwise distances for diversity metrics or abundance of PP genera were correlated with corresponding matrices of temperature differences (see Supplementary File 1 for more details). Due to the high inter-individual variability of alpha-diversity estimates observed within some dates and months, and to test whether temperature could prompt such variability, the impact of temperature on the standard deviations of Shannon (Shannon SD) and Faith’s PD (PD SD) indices was also assessed for skin and gill microbiota using the same set of tests (*lm* and PERMANOVA, as well as *gls* for pairwise matrices).

The metagenomic Phylogenetic Investigation of Communities by Reconstruction of Unobserved States software (PICRUSt2) embedded in QIIME2^54^ was used to predict the putative microbial metabolism, applying a weighted nearest taxon index (NSTI) cutoff of 0.03. Predicted metagenomes were collapsed using the Kyoto Encyclopaedia of Genes and Genomes (KEGG) Pathway metadata.^55^ We identified differentially abundant predicted metabolic pathways between cold/warm months in the skin and gill microbiota using linear discriminant analysis (LDA) in LEfSe.^56^ To this end, we used a *P* value cutoff of 0.05 and a LDA effect size cutoff of 2.^56^

A more detailed description of the “Materials and Methods” above is provided in Supplementary File 1.

## Results

### Bacterial composition and temporal dynamics of the microbiota

**Fig. 1:**
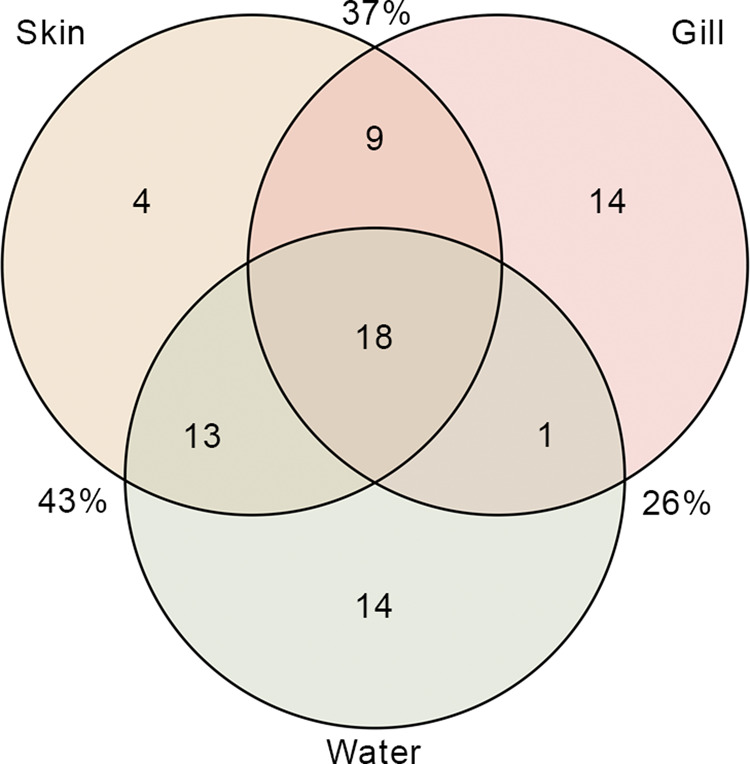
Shared core ASVs. Venn diagram showing the number and percentage of shared core ASVs between the skin and gill microbiota (N = 10 × 12 months × tissue) of the seabass *Dicentrarchus labrax* and the surrounding water (*N* = 2 × 11 months) throughout the 12 sampling months.

Six bacterial phyla and 28 genera were categorized as the most abundant taxa (≥5%) in European seabass skin and gill and associated water microbiota, throughout the 12 month study period (Figure S2, Table S1). Bacteroidota and Proteobacteria were consistently abundant across all months in the fish microbiota and water, and Verrucomicrobiota was highly abundant in the water for all months. *Glaciecola*, NS3a marine group, *Pseudoalteromonas*, *Psychrobacter*, *Rubritalea*, and an unidentified genus belonging to the Flavobacteriaceae family were the most abundant genera across all studied microbiota. However, none of the 28 genera were consistently highly abundant across all months in the fish mucosa or water. Nevertheless, there were a total of 73 core ASVs present in all three studied microbiota throughout the studied months and from these 44, 42, and 46 ASVs were present in the microbiota of the skin, gill and water, respectively (Fig. 1, Table S2). The three microbiota communities analyzed shared 25% of the core ASVs, while skin and gill shared 37%, skin and water shared 43%, and gill and water shared 26% (Fig. 1). Out of the 73 monthly core ASVs, three belonged to PP genera: a *Photobacterium* ASV in the skin, and two *Vibrio* ASVs, one present in the skin, and the other in the water (Table S2).

**Fig. 2:**
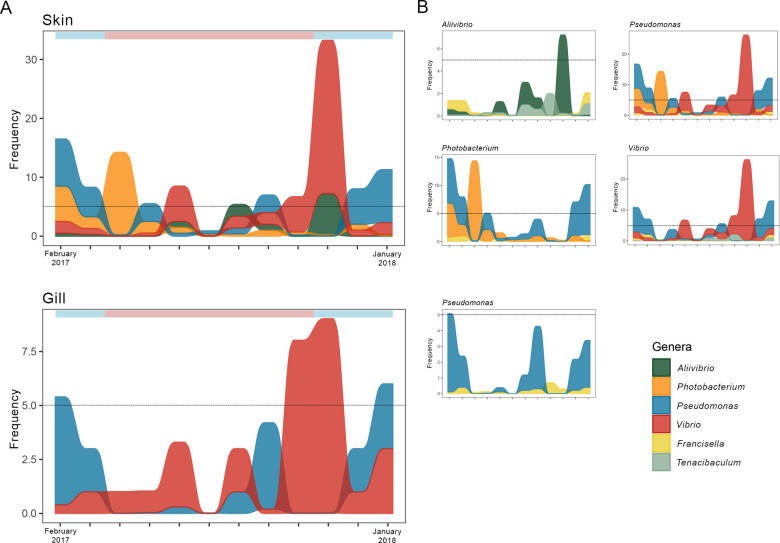
Most abundant potentially pathogenic genera. Alluvial plots of the monthly prevalence of the most abundant potentially pathogenic (PP) genera (**A**); and alluvial plots of the monthly prevalence of each PP genus and other less abundant PP genera they interact with according to TIME results (**B**). Results are depicted for the skin and gill microbiota (*N* = 10 × 12 months) of the seabass *Dicentrarchus labrax* from February 2017 (left) to January 2018 (right). Blue and red bars represent cold and warm months, respectively. Dashed line represents the 5% cutoff, above which genera were considered highly abundant.

Monthly estimates of microbial alpha-diversity of the skin and gills followed the same trends (increase/decrease) throughout the sampling period, showing significant monthly fluctuations (Kruskal–Wallis, *P* ≤ 0.05; Table S3). Pairwise comparisons showed significant shifts in alpha-diversity mainly between warm months and between cold to warm or warm to cold (hereafter termed cold/warm) transitions. Chronologically, significant decreases of Shannon and Faith’s PD indices in both fish mucosae occurred between March and April (coinciding with the transition from cold to warm months); this was followed by a significant increase of the Shannon index between April and May in the skin microbiota and a significant increase of the Faith PD index between May and June in the gill microbiota; between June and July, both alpha-diversity indices significantly decreased in both mucosae (*P* ≤ 0.05, Table S3). In addition, between October and November, significant decreases occurred in the Shannon index for the skin microbiota and in Faith’s PD for the gill microbiota, coinciding with the transition between warm to cold months (*P* ≤ 0.05, Table S3). Finally, a significant increase in the Shannon diversity occurred between November and December in the gill microbiota (*P* ≤ 0.05, Table S3). It is noteworthy that aforementioned significant decreases in alpha-diversity occurred in parallel with an increase in the abundance of some PP genera in the skin in April and in both tissues in November (Fig. 2).

**Fig. 3:**
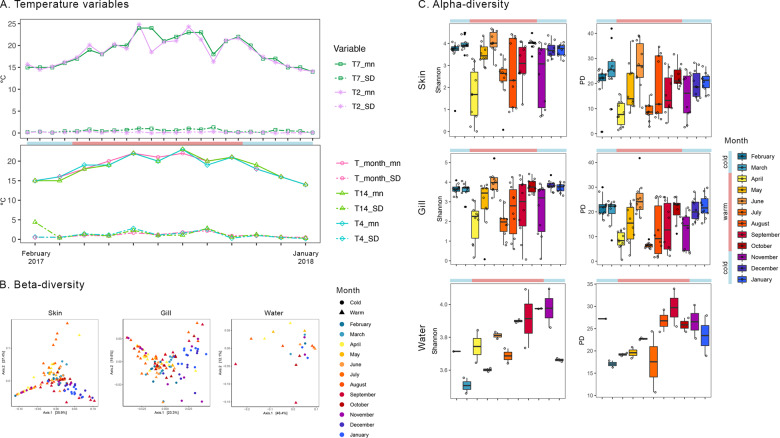
Temperature models and diversity estimates. Temperature variables (**A**), and beta-diversity (**B**) and alpha-diversity (**C**) estimates. The temperature variables were derived for each sampling date and month. Beta-diversity is represented through PCoA plots computed using Unifrac weighted distance for the skin and gill microbiota (*N* = 10 x 12 months × tissue) of the seabass *Dicentrarchus labrax* and the surrounding water (*N* = 2 × 11 months). Each dot represents a microbiota sample. Alpha-diversity estimates are represented by boxplots depicting mean values and standard deviations of Shannon and Faith’s PD estimates for the skin and gill microbiota (*N* = 10 × 12 months x tissue) of the seabass *Dicentrarchus labrax* and the surrounding water (*N* = 2 ×11 months) from February 2017 (left) to January 2018 (right). T_month_mn = mean monthly temperature; T_month_SD = standard deviation of T_month_mn; T14_mn = mean temperature from the 7 days previous to both sampling dates in a given month; T14_SD = standard deviation of T14_mn; T4_mn = mean temperature from the 2 days previous to both sampling dates in a given month; T4_SD = standard deviation of T4_mn; T7_mn = mean temperature from the 7 days previous to a sampling date; T7_SD = standard deviation of T7_mn; T2_mn = mean temperature from the 2 days previous to a sampling date; T2_SD = standard deviation of T2_mn.

Although microbial structure showed significant differences between months (PERMANOVA, *P* ≤ 0.05; Table S3), pairwise comparisons showed no significant differences between consecutive months (corrected *P* ≥ 0.05, Table S3). Moreover, there was no visible clustering of samples per month or per cold/warm months when dissimilarities were plotted in the PCoAs (Fig. 3).

**Fig. 4:**
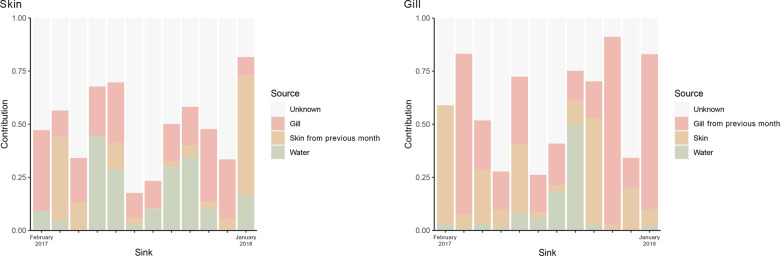
Microbiota contribution between sources and sinks. Barplots depicting the percentage of microbiota contribution from each source to the microbiota of the skin and gill of the seabass *Dicentrarchus labrax* per month (February 2017 to January 2018).

The results from the fast expectation-maximization for microbial source tracking (FEAST) showed that the dynamics of potential bacterial transferral between the different available source microbiota (i.e., skin, gill and water) and the tested sink fish mucosae (skin or gill) varied throughout the months (Fig. 4). Overall, the skin microbiota was more variable and dynamic, maintaining only a small fraction of its microbial diversity between months (mean 13 ± 18%) (Fig. 4). Unknown sources were the major contributors (mean 51 ± 19%) for the skin microbial community at each given month, followed by the gill microbiota (21 ± 9%) and the water microbiota (17 ± 15%) (Fig. 4). Overall, the gill microbiota was more stable throughout the year than the skin microbiota, with a larger fraction of its bacterial diversity (36 ± 28%) maintained between months. Similar to the skin microbiota, most of its composition was attributed to unknown sources (mean 40 ± 23%), whereas the skin microbiota (mean 19 ± 19%) and water microbiota (mean 8 ± 15%) had a lower contribution to the gill microbiota (Fig. 4).

### Dynamics of potentially pathogenic (PP) genera

Several PP genera were highly abundant (i.e., ≥5% of total sequences) in both skin and gills throughout the year, with the highest incidence of PP genera in the skin microbiota (Fig. 2, Table S1). Specifically, *Aliivibrio*, *Photobacterium*, *Pseudomonas,* and *Vibrio* were highly abundant in the skin; while *Pseudomonas* and *Vibrio* were highly abundant in the gill microbiota.

The abundance of the PP genera was correlated with the abundance of several other genera, with the exception of *Pseudomonas* in the gill microbiota (Table S4). In general, each of the PP genera detected was correlated with a higher number of other genera in the skin than in the gill (Table S4). In the skin microbiota, there were mostly negative correlations between the abundance of PP and other genera, except for *Pseudomonas*, where correlations were mostly positive. In the gills, correlations between taxa abundances were mostly positive (Table S4). The abundances of all PP genera in both tissues, except for *Vibrio* in the gill, were affected by other potentially pathogenic genera (Table S4). These interactions consisted mostly of negative correlations, and only the *Pseudomonas-Francisella* correlation was positive in both tissues (Pearson Correlation, Table S4). Interestingly, during warm months, peaks in the abundance of PP genera in the skin microbiota did not occur concomitantly, also suggesting higher competition between PP genera in these months (Fig. 2). Furthermore, in the skin microbiota, both *Halobacteriovorax* or *Sphingomonas*, which contain species with probiotic properties,^57,58^ showed a negative interaction with all but one (*Pseudomonas*) PP genera. In the gill microbiota, interactions with genera with known probiotic properties were not detected (Table S4).

### Effect of water temperature on the fish microbiota

There was a significant impact of all the tested temperature variables on at least one of the alpha-diversity metrics (*lm*, *p* ≤ 0.05, Table 1), with the exception of T2_SD in the skin and T14_SD and T7_SD in the gill microbiota (Table 1). The effect of water temperature was more noticeable in the gill microbiota compared to the skin, where the influence of water temperature was more significant in the standard deviations of the Shannon and PD metrics than on their mean values (Table 1). The influence of all temperature variables was also denoted in the UniFrac distances, with the exception of T14_SD in the skin and T4_SD in the gill (Table 1). In addition, seasonality significantly impacted the skin and gill microbiota, as seen in all models except for the gill PD SD metric when including T7 and T14 variables (*p* ≤ 0.05, Table 1). Interestingly, the phylogenetic diversity of the skin microbiota was only influenced by cold/warm months and season (Table 1).

**Table 1:** Significance of the temperature models and season on the diversity indices in the skin and gill microbiota of the seabass *Dicentrarchus labrax*.

			Shannon	Shannon SD	PD	PD SD	Unifrac We.	Unifrac Un.
Skin	Samples grouped by Month	T4_mn	2.6 (0.1)	**14.1 (0.0003)**	0.4 (0.5)	**23.7 (4**^**−6**^**)**	**0.1 (9**^**−5**^**)**	**0.04 (9**^**−5**^**)**
		T4_SD	0.3 (0.6)	**7.2 (0.01)**	0.01 (0.9)	**15.0 (0.0002)**	0.02 (0.1)	**0.01 (0.04)**
		Season	**6.6 (0.004)**	**6.3 (0.001)**	**12.4 (5**^**−7**^**)**	**5.9 (0.001)**	**0.1 (9**^**−5**^**)**	**0.1 (9**^**−5**^**)**
		T14_mn	2.4 (0.1)	**22.4 (6**^**−6**^**)**	0.6 (0.5)	**24.8 (2**^**−6**^**)**	**0.1 (9**^**−5**^**)**	**0.04 (9**^**−5**^**)**
		T14_SD	0.03 (0.9)	**18.2 (4**^**−5**^**)**	0.01 (0.9)	**6.7 (0.01)**	0.01 (0.2)	0.01 (0.05)
		Season	**6.4 (0.001)**	**7.3 (0.0002)**	**10.4 (4**^**−6**^**)**	**4.3 (0.01)**	**0.1 (9**^**−5**^**)**	**0.1 (9**^**−5**^**)**
		T_month_mn	1.4 (0.2)	**9.2 (0.003)**	0.6 (0.5)	**18.7 (3**^**−5**^**)**	**0.1 (9**^**−5**^**)**	**0.04 (9**^**−5**^**)**
		T_month_SD	2.2 (0.1)	**23.0 (5**^**−6**^**)**	0.6 (0.5)	**14.8 (0.0002)**	**0.1 (2**^**−4**^**)**	**0.01 (0.01)**
		Season	**9.3 (2**^**−5**^**)**	**6.7 (0.0003)**	**13.9 (9**^**−8**^**)**	**6.6 (0.0004)**	**0.1 (9**^**−5**^**)**	**0.1 (9**^**−5**^**)**
		Cold/Warm	**4.4 (0.04)**	**3.9 (0.04)**	**4.1 (0.04)**	1.1 (0.3)	**0.1 (9**^**−5**^**)**	**0.04 (9**^**−5**^**)**
		Season	**4.9 (0.003)**	**25.7 (9**^**−13**^**)**	**3.8 (0.01)**	**5.9 (0.001)**	**0.1 (0.003)**	**0.1 (9**^**−5**^**)**
	Biweekly data	T2_mn	**5.7 (0.02)**	**6.4 (0.01)**	3.8 (0.1)	0.04 (0.8)	**0.1 (9**^**−5**^**)**	**0.04 (9**^**−5**^**)**
		T2_SD	1.5 (0.2)	1.9 (0.2)	0.8 (0.4)	1.4 (0.2)	**0.02 (0.03)**	0.01 (0.1)
		Season	**1.5 (0.03)**	**8.2 (6**^**−5**^**)**	**3.5 (0.02)**	**5.0 (0.003)**	**0.1 (9**^**−5**^**)**	**0.1 (9**^**−5**^**)**
		T7_mn	**4.3 (0.04)**	**6.8 (0.01)**	3.4 (0.1)	0.01 (0.9)	**0.1 (9**^**−5**^**)**	**0.1 (9**^**−5**^**)**
		T7_SD	2.2 (0.1)	**10.1 (0.002)**	1.7 (0.2)	2.5 (0.1)	**0.02 (0.02)**	**0.01 (0.02)**
		Season	**2.9 (0.04)**	**5.3 (0.002)**	**3.2 (0.03)**	**5.4 (0.002)**	**0.1 (9**^**−5**^**)**	**0.1 (9**^**−5**^**)**
Gill	Samples grouped by Month	T4_mn	**7.1 (0.001)**	**59.7 (5**^**−12**^**)**	**8.9 (0.003)**	**28.2 (5**^**−7**^**)**	**0.1 (9**^**−5**^**)**	**0.03 (9**^**−5**^**)**
		T4_SD	0.3 (0.6)	**28.4 (5**^**−7**^**)**	0.2 (0.7)	**24.6 (3**^**−6**^**)**	0.01 (0.2)	0.01 (0.1)
		Season	**10.5 (4**^**−6**^**)**	**13.1 (2-**^**7**^**)**	**14.3 (6**^**−8**^**)**	**4.3 (0.01)**	**0.1 (9**^**−5**^**)**	**0.1 (9**^**−5**^**)**
		T14_mn	**6.8 (0.01)**	**75.1 (4**^**−14**^**)**	**8.4 (0.01)**	**35.1 (3**^**−8**^**)**	**0.1 (9**^**−5**^**)**	**0.04 (9**^**−5**^**)**
		T14_SD	0.1 (0.7)	2.8 (0.1)	1.9 (0.2)	2.3 (0.1)	**0.02 (0.003)**	0.01 (0.1)
		Season	**8.9 (2**^**−5**^**)**	**13.8 (9**^**−8**^**)**	**11.0 (2**^**−6**^**)**	0.8 (0.5)	**0.1 (0.0002)**	**0.1 (9**^**−5**^**)**
		T_month_mn	**7.4 (0.01)**	**70.6 (1**^**−13**^**)**	**9.5 (0.003)**	**36.6 (2**^**−8**^**)**	**0.1 (9**^**5**^**)**	**0.03 (9**^**5**^**)**
		T_month_SD	1.9 (0.2)	**55.8 (2**^**−11**^**)**	0.6 (0.4)	**75.1 (4**^**−14**^**)**	**0.02 (0.003)**	**0.01 (0.01)**
		Season	**10.6 (3**^**−6**^**)**	**10.3 (5**^**−6**^**)**	**14.5 (5**^**−8**^**)**	**9.2 (2**^**−5**^**)**	**0.1 (9**^**−5**^**)**	**0.1 (9**^**−5**^**)**
		Cold/Warm	**11.8 (0.001)**	**58.8 (6**^**−12**^**)**	**12.5 (0.001)**	**7.5 (0.01)**	**0.1 (9**^**−5**^**)**	**0.04 (9**^**−5**^**)**
		Season	**5.9 (0.001)**	**27.9 (1**^**−13**^**)**	**7.2 (0.0002)**	**3.3 (0.02)**	**0.1 (4**^**−4**^**)**	**0.1 (9**^**−5**^**)**
	Biweekly data	T2_mn	**10.3 (0.002)**	**26.0 (1**^**−6**^**)**	**13.1 (0.001)**	1.6 (0.2)	**0.1 (9**^**−5**^**)**	**0.03 (9**^**−5**^**)**
		T2_SD	0.1 (0.7)	**16.4 (10**^**−5**^**)**	0.3 (0.6)	**15.4 (0.0002)**	0.01 (0.3)	**0.01 (0.01)**
		Season	**5.4 (0.002)**	**10.3 (5**^**−6**^**)**	**5.8 (0.001)**	**2.8 (0.04)**	**0.1 (0.0002)**	**0.1 (9**^**−5**^**)**
		T7_mn	**12.9 (0.001)**	**41.0 (4**^**−9**^**)**	**15.6 (0.0001)**	0.5 (0.5)	**0.1 (9**^**−5**^**)**	**0.04 (9**^**−5**^**)**
		T7_SD	0.01 (0.9)	2.0 (0.2)	0.01 (0.9)	1.0 (0.3)	0.01 (0.1)	**0.01 (0.02)**
		Season	**10.0 (7**^**−6**^**)**	**24.2 (3**^**−12**^**)**	**10.5 (4**^**−6**^**)**	1.7 (0.2)	**0.1 (9**^**−5**^**)**	**0.1 (9**^**−5**^**)**

For each test we report F (alpha-diversity) or *R*^2^ (beta-diversity) statistic (*p*). Significant associations are indicated in bold. Models were built using: (1) samples grouped by month; and (2) biweekly samples. Three different sets of variables were built used using temperature measured at different time scales: days (T2_mn and T4_mn: mean temperature from the 2 previous days to sampling; T2_SD and T4_SD: standard deviations of mean temperatures observed in the 2 previous days to sampling); weeks (T7_mean, T14_mean and T_month_mean: etc etc); and months (cold/warm: variable with two levels built by grouping months according to the annual mean temperature observed (18.5°). Season was a categorical variable with four levels (spring, summer, autumn, winter) and used as a fixed effect in all models to account for unmeasured environmental changes that may vary seasonally.

Changes in both alpha- and beta-diversity of fish microbiota were significantly correlated with changes in all temperature variables (*P* ≤ 0.03; Table 2). Evidently, as depicted in Fig. 3, there was a positive relation between alpha-diversity and T4_SD in warm months (i.e., both values increased or decreased between months) and negative in cold months (i.e., there was an inverse relation between alpha-diversity and T4_SD).

**Table 2:** Test results from gls models indicating the significance of correlation between temperature variables and bacterial diversity, including the abundance of main (>5% of total reads) potentially pathogenic (PP) genera.

	Months							Date			
Skin	Cold/warm months	T_month mn	T_month SD	T14_mn	T14_SD	T4_mn	T4_SD	T7_mn	T7_SD	T2_mn	T2_SD
Shannon	**21 (0.0001)**	**21 (0.0001)**	**48.8 (0.0001)**	**46.6 (0.001)**	**28 (0.0001)**	**55 (0.0001)**	**96 (0.0001)**	**29 (0.0001)**	**31 (0.0001)**	**38 (0.0001)**	**7 (0.01)**
Shannon SD	**21 (0.0001)**	**102 (0.0001)**	**367 (0.0001)**	**224 (0.0001)**	**940 (0.0001)**	**183 (0.0001)**	**663 (0.0001)**	3 (0.1)	**28 (0.0001)**	**16 (1**^**–4**^**)**	**21 (0.0001)**
PD	**32 (0.0001)**	**25 (0.0001)**	**38 (0.0001)**	**45 (0.0001)**	**14 (2**^**-4**^**)**	**58 (0.0001)**	**72 (0.0001)**	**22 (0.0001)**	**36 (0.0001)**	**27 (0.0001)**	1.5 (0.2)
PD SD	**6 (0.01)**	**155 (0.0001)**	**379 (0.0001)**	**223 (0.0001)**	**233 (0.001)**	**224 (0.0001)**	**427 (0.0001)**	3 (0.1)	**28 (0.0001)**	**16 (1**^**-4**^**)**	**21 (0.0001)**
Unifrac weighted	**151 (0.0001)**	**169 (0.0001)**	**146 (0.0001)**	**164 (0.0001)**	**71 (0.0001)**	**230 (0.0001)**	**187 (0.0001)**	**191 (0.0001)**	**48 (0.0001)**	**324 (0.0001)**	**45 (0.0001)**
Unifrac unweighted	**532 (0.0001)**	**562 (0.0001)**	**450 (0.0001)**	**653 (0.0001)**	**102 (0.0001)**	**673 (0.0001)**	**369 (0.0001)**	**704 (0.0001)**	**196 (0.0001)**	**634 (0.0001)**	**18 (0.0001)**
*Aliivibrio*	0.6 (0.4)	0.8 (0.4)	**5 (0.02)**	3 (0.1)	**10 (0.002)**	1 (0.3)	**5 (0.03)**	0.9 (0.4)	**5 (0.02)**	0.9 (0.4)	2 (0.2)
*Photobacterium*	2 (0.2)	**9 (0.003)**	**4 (0.04)**	**16 (0.0001)**	**18 (0.0001)**	**7 (0.01)**	2 (0.2)	2 (0.1)	0.3 (0.6)	0.6 (0.4)	**8 (0.01)**
*Pseudomonas*	**306 (0.0001)**	**564 (0.0001)**	**248 (0.0001)**	**736 (0.0001)**	**37 (0.0001)**	**711 (0.0001)**	**90 (0.0001)**	**839 (0.0001)**	**19 (0.0001)**	**850 (0.0001)**	3 (0.1)
*Vibrio*	3 (0.1)	1 (0.3)	1 (0.3)	**5 (0.03)**	0.7 (0.4)	3 (0.1)	0.7 (0.4)	**13 (3**^**-4**^**)**	**5 (0.02)**	**9 (0.003)**	3 (0.1)
Gill											
Shannon	**87 (0.0001)**	**61 (0.0001)**	**93 (0.0001)**	**91 (0.0001)**	**33 (0.0001)**	**111 (0.0001)**	**146 (0.0001)**	**81 (0.0001)**	**17 (0.0001)**	**106 (0.0001)**	2 (0.2)
Shannon SD	**483 (0.0001)**	**908 (0.0001)**	**1886 (0.0001)**	**1379 (0.0001)**	**302 (0.0001)**	**1136 (0.0001)**	**2030 (0.0001)**	**60 (0.0001)**	**115 (0.0001)**	**67 (0.0001)**	**354 (0.0001)**
PD	**88 (0.0001)**	**76 (0.0001)**	**104 (0.0001)**	**110 (0.0001)**	**44 (0.0001)**	**129 (0.0001)**	**139 (0.0001)**	**97 (0.0001)**	**17 (0.0001)**	**115 (0.0001)**	1 (0.3)
PD SD	**182 (0.0001)**	**820 (0.0001)**	**3347 (0.0001)**	**1189 (0.0001)**	**210 (0.0001)**	**651 (0.0001)**	**1773 (0.0001)**	**60 (0.0001)**	**115 (0.0001)**	**67 (0.0001)**	**354 (0.0001)**
Unifrac weighted	**223 (0.0001)**	**230 (0.0001)**	**148 (0.0001)**	**225 (0.0001)**	**89 (0.0001)**	**265 (0.0001)**	**140 (0.0001)**	**244 (0.0001)**	**30 (0.0001)**	**255 (0.0001)**	**24 (0.0001)**
Unifrac unweighted	**624 (0.0001)**	**658 (0.0001)**	**498 (0.0001)**	**736 (0.0001)**	**103 (0.0001)**	**787 (0.0001)**	**397 (0.0001)**	**709 (0.0001)**	**134 (0.0001)**	**651 (0.0001)**	**28 (0.0001)**
*Pseudomonas*	**236 (0.0001)**	**446 (0.0001)**	**172 (0.0001)**	**461 (0.0001)**	**498 (0.0001)**	**554 (0.0001)**	**43 (0.0001)**	**720 (0.0001)**	**15 (2**^**–4**^**)**	**1014 (0.0001)**	**5 (0.03)**
*Vibrio*	0.1 (0.7)	0.02 (0.9)	0.5 (0.5)	0.5 (0.5)	3 (0.1)	0.4 (0.5)	1 (0.3)	3 (0.1)	**23 (0.0001)**	2 (0.2)	**8 (0.004)**
Water											
Shannon	0.1 (0.8)	4 (0.1)	**6 (0.02)**	**20 (0.0001)**	0.3 (0.6)	**9 (0.003)**	0.3 (0.6)	**6 (0.02)**	0.1 (0.8)	**6 (0.01)**	0.1 (0.8)
PD	0.6 (0.4)	0.03 (0.9)	1 (0.2)	0.5 (0.5)	0.6 (0.4)	0.04 (0.9)	0.04 (0.9)	0.004 (0.9)	0.0002 (0.9)	0.4 (0.6)	0.6 (0.4)
Unifrac weighted	**5 (0.03)**	3 (0.1)	3 (0.1)	4 (0.1)	1 (0.5)	4 (0.1)	1 (0.3)	2 (0.2)	1 (0.3)	2 (0.2)	0.3 (0.6)
Unifrac unweighted	**25 (0.0001)**	**29 (0.0001)**	**14 (2**^**–4**^**)**	**45 (0.0001)**	1 (0.3)	**39 (0.0001)**	**6 (0.01)**	**31 (0.0001)**	**6 (0.01)**	**35 (0.0001)**	0.1 (0.7)

Correlation structure was estimated based on distance matrices for both dependent and independent variables using the corMLPE R package. For each test we report the F statistic and significance (*P* value with significant correlations indicated in bold). Months were separated into cold and warm based on whether the mean monthly temperature was lower or higher than the daily annual average (i.e., 18.6 °C). T_month_mn: mean monthly temperature; T_month_SD: standard deviation of T_month_mn; T14_mn: mean temperature of the 7 days previous to both sampling dates in a month; T14_SD: standard deviation of T14_mn; T4_mn: mean temperature from the 2 days previous to both sampling dates in a given month; T4_SD: standard deviation of T4_mn; T7_mn: mean temperature from the 7 days previous the sampling date; T7_SD: standard deviation of T7_mn; T2_mn: mean temperature from the 2 days prior to the sampling date; T2_SD: standard deviation of T2_mn.

Overall, there was a significant correlation between changes in abundance of the PP genera in the skin and gill microbiota and changes in at least one of the temperature variables (*P* ≤ 0.05; Table 2). Variation in the abundance of *Pseudomonas* was correlated to all changes in temperature, independently of the considered time window; while changes in the abundance of *Aliivibrio* were correlated to changes to the standard deviation of the mean temperatures (Table 2). Variation in the abundance of *Photobacterium* was correlated with long-term changes in temperature, while changes in the abundance of *Vibrio* were correlated with changes in mean temperatures in the skin and standard deviations in the gill (Table 2).

**Fig. 5:**
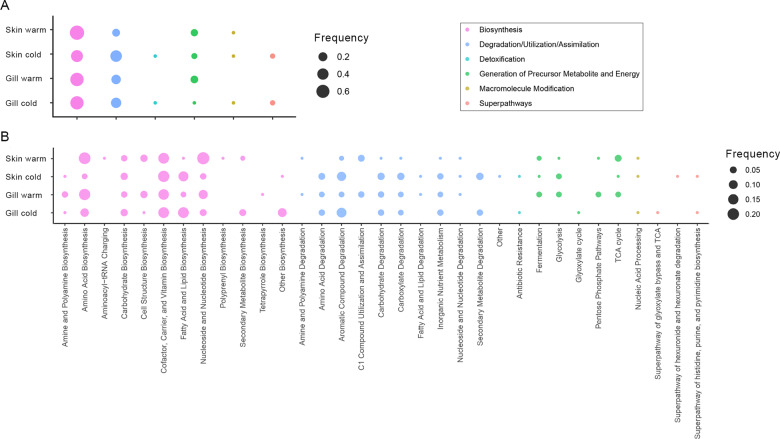
Differentially enriched potential pathways. Relative frequency of the differentially enriched potential pathways in the skin and gill microbiota of the seabass *Dicentrarchus labrax* during cold and warm months grouped by broader (**A**) or smaller (**B**) KEGG categories. Circles are colored according to broader categories.

PICRUSt2 predicted 484 and 477 KEGG pathways in the seabass skin and gill microbiota, respectively. In the skin microbiota, 84 and 80 predicted pathways were differentially abundant in warm and cold months, respectively; while 70 and 78 predicted pathways were differentially abundant in the gill microbiota during warm and cold months, respectively (Table S5). Interestingly, most of the differently abundant potential pathways were shared between the skin and gill microbiota in cold (51%) and warm (44%) months. Predicted pathways related to biosynthesis, degradation/utilization/assimilation and generation of precursor metabolite and energy were enriched in cold and warm months in the microbiota of both mucosae (Fig. 5, Table S5). Predicted pathways related to detoxification and superpathways were only enriched in cold months in both mucosae. In the skin microbiota, there was a decrease in the frequency of predicted pathways related to biosynthesis and an increase in degradation/utilization/assimilation related pathways from warm to cold months (76% to 47% and 13% to 43%, respectively) (Fig. 5, Table S5). The microbiota of both tissues showed a decrease in the frequency of predicted pathways related to generation of precursor metabolite and energy from warm to cold months (10% to 5% in the skin; 12% to 1% in the gill) (Fig. 5, Table S5).

At a finer scale, the microbiota of both tissues presented a decrease in frequency of potential pathways related to amino acid biosynthesis, cell structure biosynthesis, nucleoside and nucleotide biosynthesis, and TCA cycle from warm to cold months (Fig. 5, Table S5). In contrast, potential pathways related to fatty acid and lipid biosynthesis and aromatic compound degradation increased in frequency from warm to cold months in both tissues. In the microbiota of both tissues, amine and polyamine degradation, C1 compound utilization and assimilation and pentose phosphate predicted pathways were only enriched in warm months; whereas secondary metabolite degradation, antibiotic resistance and the super pathway of histidine, purine, and pyrimidine biosynthesis predicted pathway categories were only enriched in the cold months (Fig. 5, Table S5).

## Discussion

The skin and gill microbiota of the farmed European seabass were highly dynamic, being significantly affected by water temperature. Although previous studies reported an effect of water temperature on fish microbiota, they focused on testing the effects of single temperatures^6,18,20^ or established an indirect link with temperature based on samples taken from different geographic locations.^19,21^ To the best of our knowledge, the only other longitudinal study which specifically tested the effects of natural fluctuations of sea water temperatures on fish microbiota was by Minich et al.^9^ on chub mackerel. In that study, although temperature significantly affected the skin and gill microbial composition on each sampling date, an effect on beta-diversity was less clear.^9^ Our results show that mean water temperature and associated standard deviations occurring at different time scales, significantly affected the composition, structure and function of the skin and gill microbiota of the European seabass *Dicentrarchus labrax*. In addition, temperature seems to be a factor triggering dysbiosis.

### Temporal dynamics of the microbiota

The skin and gill microbiota of farmed European seabass varied significantly over a 12 month period, particularly at lower taxonomic levels (most abundant genera and core ASVs), while maintaining phylogenetic relatedness at higher taxonomic levels (most abundant phyla). Similar long-term patterns have been reported in the microbiota of other teleost species, including the Atlantic charr (gut;^59^), tench (gut;^60^), gulf killifish (skin;^61^) and the largemouth bass, bluegill, and spotted gar (skin and gut;^4^)

The skin and gill microbiomes of healthy European seabass can be very distinct,^37^ responding asymmetrically to disease (photobacteriousis) and with different recovery trajectories after antibiotic treatment.^62^ Here, despite such intrinsic differences, the overall bacterial composition of both mucosae followed the same trends, i.e., concomitantly increasing or decreasing in diversity throughout the year. This suggests they are likely modulated by the same host and/or environmental factors. Furthermore, our analysis showed that the dynamics of the skin and gill microbiota are complex, with only a small portion of the microbiota remaining stable over time (ca. 13% in the skin and ca. 36% in the gill on average over the 12 months). Skin and particularly the gills seem to be highly selective habitats, and bacterial variation did not seem to arise due to substantial outsourcing of bacteria from the water column. This result is in line with previous studies where water microbiota was shown to have a minor influence on the mucosal microbiota of adult fish.^3,5,11,63–65^ Instead, bacterial recruitment from the neighboring environments seems to be more important. These results suggest that there is a continuous microbial exchange of communities in the skin and gill, which could be to constant abiotic changes in the water environment (e.g., temperature) prompting complex microbial dynamics.

The high frequency of dysbiotic events, coupled with the dynamics observed in the abundances of PP genera in both fish mucosae, seems to be in line with what has been reported in farmed fish, where stress is often associated with a decrease in microbial diversity and increased susceptibility to secondary bacterial opportunists.^1,3,66^ Furthermore, the negative correlation observed between abundance of PP genera and genera with probiotic properties confirms direct competition between taxa has an important role in controlling the abundance of PP genera and potential disease outcome.^14^ Specifically, *Halobacteriovorax* and *Sphingomonas* had a negative impact on the abundance of *Aliivibrio* and *Vibrio* or *Photobacterium*, respectively, in the skin microbiota. The probiotic properties of *Halobacteriovorax* were previously demonstrated against *Vibrio parahaemolyticus* in mussels.^67^ In addition, *Sphingomonas* species were described as having antagonistic properties against the fish pathogen *Vibrio anguillarum* in roho fingerlings.^58^

### Water temperature effects in the diversity of fish microbiota

Here we show that seasonal patterns, which influence physiochemical properties of water and are highly influenced by temperature, contribute significantly to the high variability of farmed European seabass microbiota composition and structure over a year. Seasonal changes in the composition and structure of fish microbiota have been reported for the skin and gut (e.g.,^4,60^). However, such studies are commonly based on sparsely sampled timepoints (e.g., May, August and November^4^; Autumn 2017 and Summer 2018^60^). In addition, the reported effect of season in the microbiomes of wild fish is mostly linked to differences in prey availability (e.g.,^68^) or water properties like chlorophyll a, salinity and temperature (e.g.,^9^). Understanding seasonal variations in fish microbiota can be very important in an aquaculture context, for example to understand disease dynamics and outbreaks. However, the lack of longitudinal studies is still hampering such insights.

The bacterial composition and structure of farmed European seabass microbiota were significantly affected by changes in temperature occurring over both short (two days) and longer (several months) time frames. This is not surprising given the high sensitivity of bacteria to temperature.^16^ Microbial shifts occurring at small temporal scales, from just a few hours to days are not unprecedented for fish. Such events occurred, for example, after cold temperature shock (tropical tilapia^6^), acclimation (e.g., to the wild in the common snook;^29^ from freshwater to seawater in the Atlantic salmon;^69^), chemical exposure (e.g., in zebrafish;^70^ channel catfish^71^) or parasitism (e.g., in Atlantic salmon^72^). In the present work, temperature was a significant factor influencing high variation in bacterial composition within samples. Differences in bacterial diversity measures between individuals were significantly correlated with the difference in temperatures experienced by individuals across short and long time frames. In light of these results, we suggest that changes in water temperature played a major role in the high temporal variability observed in the microbiota of several teleost species (e.g.,^61,63,73^) including the European seabass.^5,37^

Another emerging trend in our analysis was that the microbiota of the European seabass was more dynamic during the warmer months and more prone to dysbiosis during cold/warm transition periods. Microbial diversity significantly decreased on three occasions during the sampled year, signaling bacterial imbalance. During cold/warm transitions, decreases in diversity were accompanied by an increase in the abundance of PP genera (i.e., *Alivibrio*, *Photobacterium* or *Vibrio*), highlighting the opportunistic nature of these bacteria. Seasonal occurrence of Vibriosis and Photobacteriosis in fish was previously observed (e.g.,^22,23^) with higher abundances of *Vibrio* and *Photobacterium* reported during warmer months, even if fish remained asymptomatic (e.g.,^9,18,20^). On the other hand, higher incidence of severe outbreaks of both *Aliivibrio* (e.g.,^74,75^) and *Pseudomonas* (e.g.,^76,77^) species usually occur at lower temperatures, explaining the high abundances of these PP genera in cold months. Temperature ranges observed here during cold/warm transition periods (15–20 °C) are known to impact European sea bass homeostasis, reducing adaptive immune response and increasing susceptibility to disease (17–23 °C;^78^). Moreover, the adaptive immune response of European seabass is intrinsically linked to temperature, being delayed at low temperatures (≤18 °C) and stimulated at higher temperatures (≥24 °C).^78,79^ It is possible that the cumulative effect of temperature changes occurring at cold/warm transition periods and changes in host homeostasis could have prompted dysbiosis. Nevertheless, dysbiotic events did not lead to visible signs of disease. Aspects of fish immunity are also correlated with age; for example, in rainbow trout, resistance to bacterial infections increases with host age.^80^ Since the European seabass included in our study were adults (26–32 months), age might explain their apparent resilience to PP genera upgrowth.

### Water temperature effects in the predicted microbiota function

Changes in microbial composition and structure between cold/warm periods were accompanied by significant changes in the predicted metabolic function of bacteria. It is important to note that these results should be interpreted with caution since PICRUSt2 analysis is biased towards human health-related microorganisms.^81^ Nevertheless, differentially enriched predicted metabolic pathways occurring in the skin and gill microbiota of the European seabass during warm periods were mostly related to biosynthesis processes, indicating high energetic expenditure. In particular, predicted pathways related to amino acid, and nucleoside and nucleotide biosynthesis are considered essential for bacterial growth,^82^ and were strongly enriched in both mucosae during this period. Similarly, more predicted pathways related to the generation of precursor metabolites and energy were enriched in both fish mucosae during warm months. During the cold period there was a shift, particularly in the skin, where biosynthesis and metabolic energy generation were reduced and degradation/utilization/assimilation predicted pathways increased by almost fourfold. In the gill microbiota, changes between differentially expressed predicted metabolic pathways were less evident, also pointing to a more stable microbiota. This suggests that broad time scale temperature shifts elicit microbial composition changes in order to harbor species with different predicted metabolic functions. Finally, it is worth noting that antimicrobial resistance (a detoxification mechanism) was significantly enriched in both tissues only in cold months. Suboptimal temperature conditions can favor the fitness of antibiotic resistant bacterial strains; for example, non-optimal temperature can increase the fitness of antibiotic resistant *Escherichia coli* (see^83^). Similarly, increased salinity and decreased pH can select for antibiotic resistant phenotypes in several bacterial food-related pathogens.^84^

## Conclusions

Changes in water temperature have a major impact on teleosts, leading to altered physiology and behavior,^85–88^ contributing to parasitism resistance and tolerance.^89^ We demonstrate here that water temperature oscillation also impacts the composition, structure and function of the skin and gill microbiota of farmed European seabass. In particular, dysbiosis seems to be more frequent in warm months and during cold/warm transitions, and the abundance of several PP genera is also affected by changes in water temperature. Furthermore, the microbiota of fish external mucosae behaved in similar ways with regards to their compositional diversity, suggesting they may be modulated by similar host and environmental stressors. These results highlight the need for further longitudinal studies examining the full thermal spectrum experienced by a species, as well as other environmental factors.

## Supplementary information

Supplementary Figures legends

Supplementary File 1

Table S1

Table S2

Table S3

Table S4

Table S5

Figure S1

Figure S2
