# Supplementary material for: Longitudinal sampling of external mucosae in farmed European seabass reveals the impact of water temperature on bacterial dynamics
Source: ISME Commun. 2021 Jun 21;1:28. doi: 10.1038/s43705-021-00019-x (PMC9723769; doi:10.1038/s43705-021-00019-x)
Supplement: Supplementary file 1 — Supplementary Figures legends [file 43705_2021_19_MOESM1_ESM.docx]

Figure S1. Daily average temperature taken throughout the sampling year. Each dot represents a day and red dots represent the sampling dates. Horizontal grey lines represent annual temperature daily average and standard deviation.

Figure S2. Most abundant phyla and genera present in the skin and gill microbiota (N=10 x 12 months x tissue) of the seabass *Dicentrarchus labrax* and surrounding water (N=2 x 11 months) from February 2017 (left) to January 2018 (right). Taxa were labelled to the lowest taxonomic level possible; u.g. = unknown genus.
