# Supplementary material for: Longitudinal sampling of external mucosae in farmed European seabass reveals the impact of water temperature on bacterial dynamics
Source: ISME Commun. 2021 Jun 21;1:28. doi: 10.1038/s43705-021-00019-x (PMC9723769; doi:10.1038/s43705-021-00019-x)
Supplement: Supplementary file 2 — Supplementary File 1 [file 43705_2021_19_MOESM2_ESM.docx]

**Supplementary File 1**

**Longitudinal sampling of external mucosae in farmed European seabass reveals complex bacterial dynamics and the role of water temperature**

Daniela Rosado, Raquel Xavier, Jo Cable, Ricardo Severino, Pedro Tarroso, Marcos Pérez-Losada

**Supplementary Material and Methods**

*Experimental design, sampling and processing*

European seabass were sampled in a Portuguese fish farm, from a rearing tank containing a single age cohort of mature adult seabass in order to reduce any possible maturation effects which are known to affect studied microbiota (e.g., Rosado et al., 2021). All fish were 26 months old at the beginning of the study and 38 months old at the last sampling date. We swabbed the right upper lateral part of the fish skin from head to tail and the right gill filaments between the first and second arch; fish were then released unharmed. Swabs and filters were immediately frozen at -20**°**C and then transported in dry ice to the CIBIO-InBIO laboratory where they were stored at -80**°**C until further processing.

*Data processing and statistical analysis*

DNA extraction kit (n=10) and PCR blanks (n=4) were used to control laboratory contamination. Additionally, four identical mock communities (ZymoBIOMICS Microbial Community DNA Standard) were sequenced and analyzed together with our fish samples. All fish and water samples were successfully amplified and sequenced.

Standard filtering parameters for DADA2 were used, with forward and reverse reads truncated at 220 nt and 200 nt respectively, and with a maximum of two expected errors per read. Default settings were also used for amplicon sequence variants (ASV) inference and chimera detection. Taxonomic inferences were made against the SSU SILVA Release 138 reference database (Quast et al., 2012). Approximately 4 336 483, 2 990 334 and 563 545 16S rRNA sequences were retrieved from the skin and gill microbiotas of commercially farmed European seabass and associated water samples, respectively. The number of sequences per sample ranged from 2 033 to 6 150 in the skin, 2 077 to 63 711 in the gill and 15 355 to 47 549 in the water samples. ASV abundances were normalized using the negative binomial distribution (McMurdie and Holmes, 2014). A midpoint rooted tree of ASVs was estimated using the Quantitative Insights Into Microbial Ecology 2 package (QIIME2; release 2019.7). After normalization and removal of non-bacterial reads, 5 559, 4 141 and 1 584 ASVs were assigned to the skin, gill and water microbiotas, respectively. ASVs present in negative controls (extraction kits and PCR) that were represented by >1% of the reads were removed from downstream analysis. These corresponded to <0.3% of the reads present in fish and water samples. Concomitantly, none of the abundant ASVs (>1%) in the fish samples were present in the controls. Diversity and bacterial abundances of the mock communities corresponded to that described by the manufacturer.

Phyla and genera with mean relative proportion ≥5% of sequence counts were considered the most abundant in each fish mucosa (skin and gills) and water for each month. To determine the core microbiota throughout the year, ASVs present in all months were identified in each mucosa and water samples.

For the fast expectation-maximization for microbial source tracking (FEAST; Shenhav et al., 2019) analysis, we used a monthly average ASV table as input. Then the skin/gill microbiota in a given month was considered as the sink with three potential environmental sources tested: i) the microbiota of the same mucosa obtained from the previous month (to infer the fraction of the microbiota that remains stable across consecutive months); ii) the alternative mucosal microbiota obtained in the same month (to infer microbial exchange between tissues); and iii) the water microbiota obtained in the same month (to infer microbial recruitment from the surrounding water). Finally, FEAST also reports the fraction of the sink microbiota that could potentially be attributed to other unknown sources.

To perform the Temporal Insights into Microbial Ecology (TIME; Baksi et al., 2018) analysis, an ASV table was used as input. Causality networks were inspected to identify specific genera that were affected by or were responsible for affecting the temporal changes of the most abundant PP genera. Secondly, Pearson correlation tests were used to determine whether there was any correlation between PP genera and the other genera; negative correlations indicating competition.

The standard deviation of Shannon (Shannon SD) and Faith’s PD (PD SD) indices were calculated using all 5 samples of a given sampling date or using all 10 samples of a given month, whether the temperature model to use was based on sampling date or monthly measurements, respectively.

To evaluate the effect of water temperature, we built four different temperature models encompassing different time windows to test which explained most of the variation in the studied microbiota. We first used a longer time frame, where a categorical model was created by dividing the year into cold (November, December, January, February, and March) and warm (April, May, June, July, August, September and October). A month was considered cold or warm whether its mean monthly temperature was lower or higher than the daily annual average (i.e., 18.6**°**C, Figure S1). The second set of models were based on intermediate time frames and included the mean monthly temperature measurements across all days of a month and their respective standard deviation (T_month_mn and T_month_SD). We also tested four shorter frame models, using the mean temperatures and their respective standard deviations from 2 and 7 days prior to each sampling date (T2_mn / T2_SD and T7_mn / T7_SD). For monthly analyses we combined these measurements for the two sampling points of a given month, in a total of 4 and 14 days per month (T4_mn / T4_SD and T14_mn / T14_SD). Linear models (*lm*) were used to assess variation in microbial composition and included each set of mean and standard deviation temperature models plus season, in order to account for seasonal variability. Season was categorized into winter (January, February, March), spring (April, May, June), summer (July, August, September), and fall (October, November, December). The final general *lm* formula was expressed as: microbial diversity ~ T_mn + T_SD + Season; or microbial diversity ~ cold/warm + Season. We also analyzed the relations between diversity and temperature in terms of distance between samples. Correlation structure between distance matrices was estimated by the generalized least squares models (*gls*) We used a maximum likelihood population effects model, as implemented in corMLPE R package (Clarke et al., 2002), to account for the lack of independence between pairwise samples in a distance matrix. Distance matrices were constructed for each continuous temperature variable, alpha-diversity measures and PP genera abundances using Euclidean distances, while the Gower’s distance (Tuerhong and Kim, 2014) was used for the categorical temperature variable (cold/warm months). For beta-diversity, we used the unifrac unweighted and weighted distance matrices.

**Bibliography**

Baksi KD, Kuntal BK, Mande SS. ‘TIME’: a web application for obtaining insights into microbial ecology using longitudinal microbiome data. Front Microbiol. 2018; 9: 36.

Clarke RT. Confidence Limits for Regression Relationships between Distance Matrices: Estimating Gene Flow with Distance. J Agric, Biol Environ Stat. 2002; 7: 361-372.

McMurdie PJ, Holmes S. Waste not, want not: why rarefying microbiome data is inadmissible. PLoS Comput Biol. 2014; 10(4): e1003531.

Quast C, Pruesse E, Yilmaz P, Gerken J, Schweer T, Yarza P *et al*. The SILVA ribosomal RNA gene database project: improved data processing and web-based tools. Nucleic Acids Res. 2012; 41(D1): D590-D596.

Rosado D, Pérez-Losada M, Pereira A, Severino R, Xavier R. Effects of aging on the skin and gill microbiota of farmed seabass and seabream. Anim Microbiome. 2021;3(1).

Shenhav L, Thompson M, Joseph TA, Briscoe L, Furman O, Bogumil D *et al*. FEAST: fast expectation-maximization for microbial source tracking. Nat Methods. 2019; 16(7): 627.

Tuerhong G, Kim SB. Gower distance-based multivariate control charts for a mixture of continuous and categorical variables. Expert Syst Appl. 2014; 41(4): 1701-1707.
