# Supplementary material for: Longitudinal sampling of external mucosae in farmed European seabass reveals the impact of water temperature on bacterial dynamics
Source: ISME Commun. 2021 Jun 21;1:28. doi: 10.1038/s43705-021-00019-x (PMC9723769; doi:10.1038/s43705-021-00019-x)
Supplement: Supplementary file 3 — Table S1 [file 43705_2021_19_MOESM3_ESM.docx]

**Table S1**: Relative mean proportions (%) of the most abundant phyla and genera (≥ 5%) in the skin and gill microbiota of the seabass *Dicentrarchus labrax* and in the water column (top, middle and bottom lines, respectively) across months. Taxa with ≥ 5% relative mean proportion in a month are indicated in bold. Unknown genera are identified as u.g.

| Phyla | Feb | Mar | Apr | May | Jun | Jul | Aug | Sep | Oct | Nov | Dec | Jan |
| --- | --- | --- | --- | --- | --- | --- | --- | --- | --- | --- | --- | --- |
| Actinobacteriota | 1 | 1 | 0.5 | 2 | 2 | **8** | 1 | 1 | 1 | 1 | 1 | 1 |
|  | - | - | - | - | - | - | - | - | - | - | - | - |
|  | - | - | - | - | - | - | - | - | - | - | - | - |
| Bacteroidota | **33** | **40** | **16** | **45** | **42** | **31** | **33** | **28** | **43** | **30** | **40** | **37** |
|  | **17** | **21** | **19** | **27** | **26** | **20** | **41** | **21** | **26** | **17** | **29** | **34** |
|  | **45** | **43** | **35** | **41** | **48** | **40** | **44** | **31** | **41** | **47** | - | **47** |
| Cyanobacteria | - | - | - | - | - | - | - | - | - | - | - | - |
|  | - | - | - | - | - | - | - | - | - | - | - | - |
|  | 0.3 | 0.2 | 0.5 | 2 | 2 | 1 | 0.4 | **5** | 1 | 1 | - | 0.4 |
| Firmicutes | 2 | 1 | **10** | **5** | 4 | 1 | **20** | 4 | 2 | 0.1 | 1 | 1 |
|  | 0.2 | 0.3 | **10** | 1 | 2 | 2 | **12** | 3 | 2 | 0.2 | 0.2 | 0.1 |
|  | 1 | 0.1 | **10** | 2 | 0.1 | 2 | 0.1 | 4 | 0.04 | 0.1 | - | 0.1 |
| Proteobacteria | **49** | **39** | **63** | **33** | **36** | **28** | **39** | **52** | **37** | **61** | **39** | **41** |
|  | **38** | **41** | **35** | **43** | **40** | **16** | **36** | **62** | **51** | **63** | **47** | **43** |
|  | **36** | **45** | **42** | **37** | **38** | **44** | **39** | **51** | **43** | **40** | - | **39** |
| Verrucomicrobiota | **7** | **10** | **5** | **10** | **8** | **25** | 2 | 2 | **7** | 3 | 4 | 4 |
|  | **26** | **20** | **31** | **24** | **16** | **56** | 3 | 1 | **11** | **8** | **6** | 4 |
|  | **12** | **9** | **10** | **14** | **8** | **10** | **11** | **6** | **11** | **7** | - | **9** |
| Genera |  |  |  |  |  |  |  |  |  |  |  |  |
| *Aliivibrio* | 0.5 | 0.3 | 0.01 | 0.03 | 1 | 0.003 | 2 | 1 | 0.01 | **7** | 0.04 | 0.1 |
|  | - | - | - | - | - | - | - | - | - | - | - | - |
|  | - | - | - | - | - | - | - | - | - | - | - | - |
| *Bacillus* | - | - | - | - | - | - | - | - | - | - | - | - |
|  | 0.04 | 0.04 | **10** | 0.2 | 0.03 | 1 | **11** | 2 | 0.1 | 0.1 | 0.1 | 0.03 |
|  | - | - | - | - | - | - | - | - | - | - | - | - |
| *Cobetia* | - | - | - | - | - | - | - | - | - | - | - | - |
|  | 0 | 0 | 0 | 0 | 0.01 | 0 | 0 | **10** | 1 | **12** | 0 | 0 |
|  | - | - | - | - | - | - | - | - | - | - | - | - |
| *Escherichia/Shigella* | **6** | 0.01 | 0.003 | 0.02 | 0 | 0.02 | 0.002 | 0.04 | 0.03 | 0.01 | 0.002 | 0.01 |
|  | - | - | - | - | - | - | - | - | - | - | - | - |
|  | - | - | - | - | - | - | - | - | - | - | - | - |
| *Glaciecola* | 3 | 3 | 0.2 | 1 | 3 | 0.4 | 2 | 4 | 3 | 2 | **8** | 3 |
|  | 1 | 2 | 0.3 | 1 | 2 | 0.3 | 1 | **7** | 2 | 2 | **5** | 1 |
|  | 4 | 3 | 0.2 | 1 | 4 | 2 | 3 | **8** | **5** | 4 | - | **6** |
| *Kocuria* | 0 | 0.002 | 0.001 | 0 | 0.02 | **6** | 1 | 0.01 | 0.02 | 0.01 | 0.01 | 0.01 |
|  | - | - | - | - | - | - | - | - | - | - | - | - |
|  | - | - | - | - | - | - | - | - | - | - | - | - |
| *Litoreibacter* | - | - | - | - | - | - | - | - | - | - | - | - |
|  | - | - | - | - | - | - | - | - | - | - | - | - |
|  | **5** | **5** | **5** | 4 | 2 | 2 | 2 | 1 | 3 | 4 | - | 3 |
| *Lysinibacillus* | 0 | 0 | **6** | 0 | 0 | 0 | 0 | 0 | 0.005 | 0 | 0.001 | 0 |
|  | - | - | - | - | - | - | - | - | - | - | - | - |
|  | - | - | - | - | - | - | - | - | - | - | - | - |
| *Marinobacterium* | - | - | - | - | - | - | - | - | - | - | - | - |
|  | - | - | - | - | - | - | - | - | - | - | - | - |
|  | 0.4 | 0.1 | 0.2 | 2 | **6** | 4 | 4 | **5** | **7** | 4 | - | 4 |
| *Marinomonas* | 0 | 0.004 | 0.2 | 0 | 0.01 | 0 | 0.01 | 0.01 | 0 | **5** | 0 | 0 |
|  | - | - | - | - | - | - | - | - | - | - | - | - |
|  | - | - | - | - | - | - | - | - | - | - | - | - |
| *Methylobacterium-Methylorubrum* | 0 | 0.01 | 0 | 0.004 | 0 | 0.003 | 0 | **12** | 0.05 | 0 | 0.01 | 0 |
|  | 0 | 0 | 0 | 0 | 0 | 0.001 | 0.002 | **15** | 0 | 0 | 0 | 0 |
|  | - | - | - | - | - | - | - | - | - | - | - | - |
| NS3a marine group | **10** | **11** | **5** | **14** | **11** | **6** | **10** | **8** | **9** | **8** | **10** | **10** |
|  | 4 | **7** | **6** | **10** | **6** | 3 | **10** | 3 | **6** | 3 | **7** | **5** |
|  | **17** | **16** | **7** | **13** | **15** | **10** | **12** | **7** | **10** | **12** | - | **15** |
| *Paucibacter* | - | - | - | - | - | - | - | - | - | - | - | - |
|  | 0 | 0 | 0.1 | 0.01 | 0.002 | 0.2 | **7** | 0.2 | 0 | 0 | 0.01 | 0 |
|  | - | - | - | - | - | - | - | - | - | - | - | - |
| *Photobacterium* | **6** | 2 | **14** | 2 | 1 | 0.3 | 0.4 | 1 | 0.5 | 0.2 | 1 | 0.2 |
|  | - | - | - | - | - | - | - | - | - | - | - | - |
|  | - | - | - | - | - | - | - | - | - | - | - | - |
| *Polaribacter* | 2 | **5** | 1 | 3 | 1 | **5** | **5** | 1 | 1 | 1 | 2 | **5** |
|  | - | - | - | - | - | - | - | - | - | - | - | - |
|  | 2 | 4 | 3 | 1 | 1 | 1 | 3 | 1 | 2 | 1 | - | **6** |
| *Polynucleobacter* | - | - | - | - | - | - | - | - | - | - | - | - |
|  | **7** | **11** | 3 | 4 | 3 | 1 | 3 | 4 | 3 | 3 | **7** | **7** |
|  | - | - | - | - | - | - | - | - | - | - | - | - |
| *Pseudoalteromonas* | 0.1 | 0.2 | **5** | 0.1 | 1 | 0.01 | 1 | 1 | 1 | **6** | 0.2 | 1 |
|  | 0.04 | 0.01 | 0.05 | **10** | 1 | 0.02 | 0.1 | 1 | 0.4 | **17** | 0.2 | 0.1 |
|  | 0 | **9** | **13** | 3 | 0.01 | 0.2 | 0.3 | 1 | 0.1 | 0.03 | - | 0.5 |
| *Pseudomonas* | **8** | **5** | 0.2 | 3 | 0.5 | 0.5 | 1 | 3 | 0.2 | 0.04 | **6** | **9** |
|  | **5** | 2 | 0.01 | 0.04 | 0.3 | 0.01 | 1 | 4 | 0.02 | 0.02 | 2 | 3 |
|  | - | - | - | - | - | - | - | - | - | - | - | - |
| *Psychrobacter* | 0.1 | 0.01 | **35** | 0.01 | 0.04 | **10** | **18** | 4 | 0.04 | 0.03 | 0.02 | 0.2 |
|  | 0 | 0 | **17** | 0.01 | 0.01 | 0.4 | 0.02 | 0.1 | 0.2 | 0.3 | 0.001 | 0.03 |
|  | 0.1 | 0.03 | 2 | 2 | 0 | **11** | 0 | **11** | 0.02 | 0 | - | 0 |
| *Rubritalea* | **6** | **7** | **5** | **9** | **5** | **23** | 1 | 0.2 | 4 | 2 | 2 | 3 |
|  | **25** | **18** | **30** | **23** | **14** | **49** | 2 | 0.2 | **7** | **5** | 3 | 3 |
|  | **10** | **7** | **9** | **14** | **6** | **9** | 3 | 0.4 | 4 | **5** | - | **8** |
| *Staphylococcus* | 2 | 0 | 1 | 0 | 0.01 | 0 | **15** | 0.3 | 0.01 | 0 | 0.003 | 0.04 |
|  | - | - | - | - | - | - | - | - | - | - | - | - |
|  | - | - | - | - | - | - | - | - | - | - | - | - |
| *Vibrio* | 2 | 1 | 0.02 | 0.5 | **6** | 0.1 | 2 | 2 | **6** | **26** | 1 | 2 |
|  | 0.4 | 1 | 1 | 1 | 3 | 0.01 | 2 | 0.2 | **8** | **9** | 1 | 3 |
|  | - | - | - | - | - | - | - | - | - | - | - | - |
| *Yoonia-Loktanella* | - | - | - | - | - | - | - | - | - | - | - | - |
|  | - | - | - | - | - | - | - | - | - | - | - | - |
|  | 4 | 3 | **5** | 4 | 1 | 0 | 0 | 1 | 0.1 | 1 | - | **5** |
| *Cryomorphaceae* (u.g.) | 3 | 4 | 0.3 | 3 | 3 | 0.5 | 1 | 1 | 4 | 4 | **5** | 3 |
|  | - | - | - | - | - | - | - | - | - | - | - | - |
|  | **9** | **8** | 2 | 3 | 4 | 3 | 2 | **5** | **6** | 4 | - | **8** |
| *Flavobacteriaceae* (u.g.) | **12** | **11** | **5** | **11** | **7** | **15** | **5** | 3 | **8** | **9** | **10** | **8** |
|  | 3 | 4 | 4 | **8** | **6** | **5** | **17** | 1 | **5** | 4 | **5** | 2 |
|  | **10** | **9** | **10** | **10** | **12** | **7** | **11** | **5** | **10** | **13** | - | **7** |
| *Fokiniaceae* (u.g.) | - | - | - | - | - | - | - | - | - | - | - | - |
|  | 3 | **5** | 2 | 1 | 1 | 0.4 | 1 | 1 | 1 | 2 | 3 | 3 |
|  | - | - | - | - | - | - | - | - | - | - | - | - |
| *Rhodobacteraceae* (u.g.) | - | - | - | - | - | - | - | - | - | - | - | - |
|  | - | - | - | - | - | - | - | - | - | - | - | - |
|  | 4 | 3 | 4 | **6** | **5** | 3 | 3 | **5** | **6** | 4 | - | 4 |
| *Bacteroidia* (u.g.) | - | - | - | - | - | - | - | - | - | - | - | - |
|  | 2 | 4 | 3 | 3 | 3 | **8** | 4 | **8** | **5** | **6** | **9** | **15** |
|  | - | - | - | - | - | - | - | - | - | - | - | - |
