# Supplementary material for: Longitudinal sampling of external mucosae in farmed European seabass reveals the impact of water temperature on bacterial dynamics
Source: ISME Commun. 2021 Jun 21;1:28. doi: 10.1038/s43705-021-00019-x (PMC9723769; doi:10.1038/s43705-021-00019-x)
Supplement: Supplementary file 4 — Table S2 [file 43705_2021_19_MOESM4_ESM.docx]

**Table S2**: Core ASVs present in the skin and gill microbiota of the seabass Dicentrarchus labrax and in the surrounding water across months. Unknown genera are identified as u.g.

| Taxonomy | Skin | Gill | Water |
| --- | --- | --- | --- |
| *Aureimarina* | ASV11 | ASV11 | ASV11 |
| *Bacillus* | ASV29 | ASV29 | - |
| *Candidatus Actinomarina* | ASV165 | - | ASV165 |
| *Candidatus Aquiluna* | ASV96 | - | ASV96 |
| *Candidatus Fritschea* | - | ASV66 | - |
| *Candidatus Megaira* | - | ASV126 | - |
| *Candidatua Puniceispirillum* | - | - | ASV336 |
| *Castellaniella* | - | ASV225 | - |
| *Crocinitomix* | ASV77 | - | ASV74 |
| *Fluviicola* | ASV181 | - | ASV181 |
| *Formosa* | - | - | ASV144 |
| *Glaciecola* | ASV6 | ASV6 | ASV6 |
| *Kordiimonas* | - | - | ASV56 |
| *Litoreibacter* | ASV13 | ASV13 | ASV13 |
| *Litoricola* | - | ASV140 | ASV140 |
| *Litorimicrobium* | ASV60, ASV74, ASV88 | ASV60 | ASV88 |
| *Lutimonas* | ASV315 | - | - |
| *Marinobacterium* | ASV20 | ASV20 | ASV20 |
| MD3-55 | ASV32 | ASV32, ASV45 | - |
| NS3a marine group | ASV3, ASV7 | ASV3, ASV7 | ASV3, ASV7 |
| NS4 marine group | ASV198 | - | - |
| NS5 marine group | ASV40 | ASV40 | ASV212 |
| *Paucibacter* | ASV123 | - | - |
| *Photobacterium* | ASV14 | - | - |
| *Planktomarina* | - | - | ASV72 |
| *Planktotalea* | ASV18 | ASV18 | ASV18 |
| *Polaribacter* | ASV9, ASV87 | ASV9 | ASV9, ASV87 |
| *Polynucleobacter* | ASV16, ASV25, ASV44 | ASV16, ASV25, ASV44 | - |
| *Pseudoalteromonas* | - | ASV8 | - |
| *Pseudohongiella* | ASV38 | ASV38 | ASV38 |
| *Roseibacillus* | ASV8 | ASV77 | ASV77 |
| *Rubritalea* | ASV1 | ASV1 | ASV1 |
| SAR92 clade | ASV171 | - | ASV171 |
| *Sulfurimonas* | - | - | ASV85 |
| *Sulfurovum* | ASV47, ASV100 | ASV47, ASV100 | ASV47, ASV100, ASV195 |
| *Synechococcus* CC9902 | - | - | ASV75 |
| *Vibrio* | ASV55 | - | ASV55 |
| *Vicingus* | - | - | ASV361 |
| *Amoebophilaceae* (u.g.) | - | ASV147 | - |
| *Arcobactereaceae* (u.g.) | - | ASV139 | - |
| *Bacteroidia* (u.g.) | ASV4 | ASV4, ASV109 | ASV209 |
| *Burkholderiales Incertae Sedis* (u.g.) | - | ASV63 | - |
| *Cellvibrionales* (u.g.) | - | - | ASV26 |
| *Crocinitomicaceae* (u.g.) | ASV41 | ASV41 | ASV41 |
| *Cryomorphaceae* (u.g.) | ASV15 | - | ASV15, ASV125 |
| *Flavobacteriaceae* (u.g.) | ASV2 | ASV2 | ASV2 |
| *Halieaceae* (u.g.) | ASV26 | ASV26 | - |
| *Kordiimonadales* (u.g.) | ASV28 | ASV28 | ASV28 |
| *Marinimicrobia* (SAR406 clade) (u.g.) | - | - | ASV274 |
| *Microbacteriaceae* (u.g.) | - | ASV79 | - |
| NS11-12 marine group (u.g.) | - | - | ASV135 |
| NS9 marine group (u.g.) | ASV124 | - | ASV124 |
| *Paracaedibacteraceae* (u.g.) | - | ASV35, ASV54 | - |
| *Proteobacteria* (u.g.) | - | ASV161 | - |
| *Rhodobacteraceae* (u.g.) | ASV22, ASV52, ASV169 | ASV22, ASV69 | ASV22, ASV52, ASV169 |
| *Rickettsiaceae* (u.g.) | - | ASV51 | - |
| SAR86 clade (u.g.) | ASV131 | - | ASV131, ASV164 |
