# Supplementary material for: Longitudinal sampling of external mucosae in farmed European seabass reveals the impact of water temperature on bacterial dynamics
Source: ISME Commun. 2021 Jun 21;1:28. doi: 10.1038/s43705-021-00019-x (PMC9723769; doi:10.1038/s43705-021-00019-x)
Supplement: Supplementary file 5 — Table S3 [file 43705_2021_19_MOESM5_ESM.docx]

**Table S3**: Alpha- and beta-diversity comparisons for the skin and gill microbiota of the seabass Dicentrarchus labrax across consecutive months. For each Kruskall-Walis test (alpha-diversity) we report the chi-squared value (overall) and significance (P value, overall and pairwise) and for each PERMANOVA test (beta-diversity) we report the R2 statistics and significance (P value). Significant differences are indicated in bold.

|  |  | Shannon | PD | Unifrac weighted | Unifrac unweighted |
| --- | --- | --- | --- | --- | --- |
| Skin | Overall | **50 (6^-7^)** | **43 (1^-5^)** | **0.4 (9^-5^)** | **0.2 (9^-5^)** |
|  | Feb – Mar | 0.1 | 0.2 | 0.04 (1) | 0.04 (1) |
|  | Mar – Apr | **0.0003** | **0.001** | 0.4 (0.1) | 0.4 (0.1) |
|  | Apr – May | **0.001** | **0.05** | 0.4 (0.1) | 0.2 (1) |
|  | May – Jun | 0.1 | 0.2 | 0.2 (1) | 0.1 (1) |
|  | Jun – Jul | **0.0004** | **0.001** | 0.3 (0.1) | 0.4 (0.1) |
|  | Jul – Aug | 0.9 | 0.4 | 0.3 (0.7) | 0.1 (1) |
|  | Aug – Sep | 0.8 | 0.8 | 0.3 (0.8) | 0.02 (1) |
|  | Sep – Oct | 0.1 | 0.2 | 0.4 (0.1) | 0.3 (0.2) |
|  | Oct – Nov | **0.01** | 0.3 | 0.2 (1) | 0.1 (1) |
|  | Nov – Dec | 0.3 | 0.4 | 0.3 (0.1) | 0.1 (1) |
|  | Dec – Jan | 0.9 | 0.7 | 0.2 (1) | 0.04 (1) |
| Gill | Overall | **50 (7^-7^)** | **50 (5^-7^)** | **0.3 (9^-5^)** | **0.2 (9^-5^)** |
|  | Feb – Mar | 1 | 0.9 | 0.2 (1) | 0.05 (1) |
|  | Mar – Apr | **0.0002** | **0.001** | 0.2 (0.7) | 0.3 (0.1) |
|  | Apr – May | 0.1 | 0.1 | 0.1 (1) | 0.1 (1) |
|  | May – Jun | 0.1 | **0.04** | 0.2 (0.5) | 0.1 (1) |
|  | Jun – Jul | **0.0001** | **0.0001** | 0.1 (1) | 0.6 (0.1) |
|  | Jul – Aug | 0.3 | 0.5 | 0.1 (1) | 0.1 (1) |
|  | Aug – Sep | 0.8 | 0.9 | 0.1 (1) | 0.02 (1) |
|  | Sep – Oct | 0.3 | 0.3 | 0.1 (1) | 0.2 (1) |
|  | Oct – Nov | 0.1 | **0.03** | 0.1 (1) | 0.1 (1) |
|  | Nov – Dec | **0.03** | 0.1 | 0.2 (1) | 0.1 (1) |
|  | Dec – Jan | 0.6 | 0.6 | 0.1 (1) | 0.03 (1) |
