# Supplementary material for: Longitudinal sampling of external mucosae in farmed European seabass reveals the impact of water temperature on bacterial dynamics
Source: ISME Commun. 2021 Jun 21;1:28. doi: 10.1038/s43705-021-00019-x (PMC9723769; doi:10.1038/s43705-021-00019-x)
Supplement: Supplementary file 6 — Table S4 [file 43705_2021_19_MOESM6_ESM.docx]

**Table S4**: TIME results showing the dynamics of the most abundant (MA) potentially pathogenic (PP) genera in the skin and gill microbiota of the seabass *Dicentrarchus labrax*. Most abundant PP genera found across months and the other genera whose abundance is being influenced by (out) or influences (in) their abundance are represented. Pearson correlation coefficients (Corr. Coef.) are included. PP genera are denoted with an asterisk (*) and genera with probiotic properties are denoted with a plus (^+^).

| Tissue | MA PP genera | Other genera | Out | In | Corr. Coef. |
| --- | --- | --- | --- | --- | --- |
| Skin | *Aliivibrio* | *Alkalimarinus* | x |  | -0.3 |
|  |  | *Alteromonas* |  | x | -0.1 |
|  |  | *Arcticiflavibacter* | x |  | -0.3 |
|  |  | *Bacteroides* | x |  | -0.1 |
|  |  | *Balneola* | x | x | 0.8 |
|  |  | *Candidatus Branchiomonas* | x |  | -0.1 |
|  |  | *Candidatus Gortzia* |  | x | -0.1 |
|  |  | *Catenococcus* |  | x | -0.1 |
|  |  | *Cyanobium* PCC-6307 |  | x | -0.2 |
|  |  | *Defluviitaleaceae* UCG-011 | x |  | -0.2 |
|  |  | *Endozoicomonas* |  | x | -0.2 |
|  |  | *Francisella** | x |  | -0.2 |
|  |  | *Fusobacterium* |  | x | -0.2 |
|  |  | *Glaciecola* | x |  | 0.001 |
|  |  | *Halobacteriovorax^+^* |  | x | -0.2 |
|  |  | *Halomonas* |  | x | -0.03 |
|  |  | *Hellea* |  | x | -0.1 |
|  |  | *Lentimonas* |  | x | 0.2 |
|  |  | *Methylobacterium-Methylorubrum* |  | x | -0.04 |
|  |  | *Oleispira* |  | x | 0.2 |
|  |  | *Paeniglutamicibacter* | x |  | -0.3 |
|  |  | *Paracoccus* |  | x | -0.2 |
|  |  | *Planococcus* |  | x | -0.3 |
|  |  | *Portibacter* |  | x | -0.2 |
|  |  | *Poseidonibacter* |  | x | -0.1 |
|  |  | *Psychromonas* | x |  | -0.2 |
|  |  | *Rhodopirellula* | x |  | -0.2 |
|  |  | Sva0081 sediment group | x |  | -0.2 |
|  |  | *Tenacibaculum** |  | x | -0.03 |
|  |  | *Thalassotalea* |  | x | -0.04 |
|  | *Photobacterium* | *Acholeplasma* | X |  | -0.1 |
|  |  | *Alkalimarinus* |  | x | -0.3 |
|  |  | *Amylibacter* | X | x | 0.7 |
|  |  | *Aureispira* |  | x | -0.2 |
|  |  | *Candidatus Branchiomonas* |  | x | -0.1 |
|  |  | *Cetobacterium* | X |  | -0.2 |
|  |  | *Clostridium sensu stricto* | X |  | -0.1 |
|  |  | *Defluviitaleaceae* UCG-011 |  | x | -0.2 |
|  |  | *Desulforhopalus* | X |  | -0.3 |
|  |  | *Devosia* | X |  | -0.02 |
|  |  | *Fransicella** |  | x | -0.1 |
|  |  | *Hypnocyclicus* | X |  | -0.2 |
|  |  | *Ilumatobacter* | X |  | -0.01 |
|  |  | *Labilibacter* |  | x | -0.1 |
|  |  | *Lentisphaera* |  | x | -0.1 |
|  |  | *Lewinella* | X |  | -0.2 |
|  |  | *Litoricola* | X |  | -0.05 |
|  |  | *Maribacter* | X |  | -0.3 |
|  |  | *Marinobacter* | X |  | -0.1 |
|  |  | *Marinoscillum* | X |  | -0.2 |
|  |  | *Massilia* |  | x | 0.04 |
|  |  | MD3-55 | X |  | 0.03 |
|  |  | *Moritella* | X |  | -0.2 |
|  |  | OM43 clade | X |  | -0.03 |
|  |  | *Pantoea* | X |  | -0.1 |
|  |  | *Parahaliea* | X |  | -0.2 |
|  |  | *Phaeocystidibacter* |  | x | -0.2 |
|  |  | *Propionigenium* |  | x | -0.2 |
|  |  | *Pseudochrobactrum* | X |  | -0.1 |
|  |  | *Pseudofulvibacter* | X | x | 0.1 |
|  |  | *fffPseudomonas** |  | x | -0.2 |
|  |  | *Psychrilyobacter* |  | x | -0.1 |
|  |  | *Rhodopirellula* |  | x | -0.2 |
|  |  | RS62 marine group |  | x | 0.3 |
|  |  | *Salinirepens* | X |  | -0.2 |
|  |  | *Sanguibacter* | X |  | -0.02 |
|  |  | SAR92 clade |  | x | -0.2 |
|  |  | *Sediminispirochaeta* |  | x | -0.2 |
|  |  | SEEP-SRB1 | X |  | -0.1 |
|  |  | Sphingomonas*^+^* |  | x | -0.03 |
|  |  | Sulfurimonas |  | x | -0.2 |
|  |  | *Sulfurovum* | X |  | -0.2 |
|  |  | *Thiomicrorhabdus* |  | x | -0.2 |
|  |  | *Ulvibacter* | X |  | -0.1 |
|  |  | Wenyingzhuangia |  | x | -0.2 |
|  | *Pseudomonas* | *Alkalimarinus* | x |  | 0.7 |
|  |  | *Bacteroides* |  | x | 0.1 |
|  |  | BD1-7 clade | x |  | 0.2 |
|  |  | *Candidatus Fritschea* | x |  | 0.7 |
|  |  | *Candidatus Omnitrophus* |  | x | -0.04 |
|  |  | *Defluviitaleaceae* UCG-011 | x |  | 0.6 |
|  |  | *Desulfobulbus* | X |  | -0.3 |
|  |  | *Desulforhopalus* | x |  | 0.2 |
|  |  | *Francisella** | x |  | 0.8 |
|  |  | *Marinomonas* |  | X | -0.3 |
|  |  | *Micrococcus* |  | X | -0.1 |
|  |  | *Muricauda* |  | X | -0.1 |
|  |  | *Phaeocystidibacter* | x |  | 0.6 |
|  |  | *Photobacterium** | x |  | -0.2 |
|  |  | *Psychrilyobacter* | X |  | 0.2 |
|  |  | RBG-16-49-21 |  | X | 0.1 |
|  |  | Sva0081 sediment group |  | x | 0.2 |
|  |  | *Thiomicrorhabdus* | X |  | 0.4 |
|  |  | *Vibrio** |  | x | -0.3 |
|  | *Vibrio* | *Alkalimarinus* | X |  | -0.2 |
|  |  | *Alteromonas* |  | X | -0.1 |
|  |  | *Arcticiflavibacter* | X |  | -0.2 |
|  |  | *Bacteroides* | X |  | -0.1 |
|  |  | *Balneola* | x | X | 0.8 |
|  |  | *Candidatus Branchiomonas* | X |  | -0.1 |
|  |  | *Candidatus Gortzia* |  | X | 0.1 |
|  |  | *Candidatus Omnitrophus* |  | X | 0.7 |
|  |  | *Catenococcus* |  | x | -0.2 |
|  |  | *Citreitalea* | X |  | -0.2 |
|  |  | *Defluviitaleaceae* UCG-011 | X |  | -0.1 |
|  |  | *Endozoicomonas* |  | X | -0.1 |
|  |  | *Enterovibrio* |  | X | -0.1 |
|  |  | *Francisella** | x |  | -0.2 |
|  |  | *Fusobacterium* |  | X | -0.2 |
|  |  | *Glaciecola* | X |  | 0.02 |
|  |  | *Halobacteriovorax^+^* |  | x | -0.1 |
|  |  | *Halomonas* |  | X | -0.1 |
|  |  | *Hellea* |  | X | -0.2 |
|  |  | *Lentimonas* |  | X | 0.3 |
|  |  | *Maritimimonas* |  | X | -0.2 |
|  |  | *Methylobacterium-Methylorubrum* |  | x | -0.1 |
|  |  | *Muricauda* | X | X | 0.5 |
|  |  | *Oleispira* |  | X | 0.2 |
|  |  | *Paeniglutamicibacter* | X |  | -0.2 |
|  |  | *Paracoccus* |  | X | -0.2 |
|  |  | *Planococcus* |  | X | -0.2 |
|  |  | *Portibacter* |  | x | -0.1 |
|  |  | *Poseidonibacter* |  | x | -0.03 |
|  |  | *Propionigenium* | X |  | -0.2 |
|  |  | *Pseudomonas** | X |  | -0.3 |
|  |  | *Psychrilyobacter* | X |  | -0.1 |
|  |  | *Psychromonas* | X |  | -0.2 |
|  |  | *Rhodopirellula* | X |  | -0.2 |
|  |  | Sva0081 sediment group | X |  | -0.1 |
|  |  | *Tenacibaculum** |  | X | -0.1 |
|  |  | *Thalassotalea* |  | X | -0.1 |
|  |  | *Variovorax* |  | x | -0.1 |
|  |  | *Wenyingzhuangia* | X |  | -0.1 |
| Gill | *Pseudomonas* | *Candidatus Puniceispirillum* | X |  | 0.1 |
|  |  | *Francisella** | X |  | 0.1 |
|  |  | *Psychrilyobacter* | x |  | -0.01 |
|  | *Vibrio* | *Caedibacter* | X |  | 0.04 |
|  |  | *Candidatus Branchiomonas* | X |  | -0.03 |
|  |  | *Glaciecola* |  | X | 0.02 |
|  |  | *Labilibacter* |  | X | 0.6 |
|  |  | *Thalassotalea* | x |  | 0.7 |
|  |  | *Winogradskyella* |  | x | 0.3 |
