# Supplementary material for: Longitudinal sampling of external mucosae in farmed European seabass reveals the impact of water temperature on bacterial dynamics
Source: ISME Commun. 2021 Jun 21;1:28. doi: 10.1038/s43705-021-00019-x (PMC9723769; doi:10.1038/s43705-021-00019-x)
Supplement: Supplementary file 7 — Table S5 [file 43705_2021_19_MOESM7_ESM.docx]

**Table S5**: Significantly enriched potential pathways in the warm and cold months in the skin and gill microbiota of the seabass Dicentrarchus labrax grouped into the KEGG L1 and L2 categories. LEfSe tests were performed with a P value and LDA score cut-offs of 0.05 and of 2, respectively.

|  |  | Skin | | Gill | |
| --- | --- | --- | --- | --- | --- |
| L1 | L2 | Warm | Cold | Warm | Cold |
| Biosynthesis | Amine and Polyamine Biosynthesis | 0 | 1 (1%) | 3 (4%) | 1 (1%) |
|  | Amino Acid Biosynthesis | 18 (21%) | 2 (3%) | 13 (19%) | 7 (9%) |
|  | Aminoacyl-tRNA Charging | 1 (1%) | - | - | - |
|  | Carbohydrate Biosynthesis | 3 (4%) | 5 (6%) | 3 (4%) | 5 (6%) |
|  | Cell Structure Biosynthesis | 5 (6%) | - | 4 (6%) | 1 (1%) |
|  | Cofactor, Carrier, and Vitamin Biosynthesis | 14 (17%) | 15 (19%) | 11 (16%) | 13 (17%) |
|  | Fatty Acid and Lipid Biosynthesis | 1 (1%) | 11 (14%) | 2 (3%) | 13 (17%) |
|  | Nucleoside and Nucleotide Biosynthesis | 19 (23%) | 3 (4%) | 8 (11%) | 4 (5%) |
|  | Polyprenyl Biosynthesis | 1 (1%) | - | - | - |
|  | Secondary Metabolite Biosynthesis | 2 (2%) | - | - | 4 (5%) |
|  | Tetrapyrrole Biosynthesis | - | - | 1 (1%) | - |
|  | Other Biosynthesis | - | 1 (1%) | - | 1 (1%) |
| Degradation/  Utilization/  Assimilation | Amine and Polyamine Degradation | 1 (1%) | - | 1 (1%) | - |
|  | Amino Acid Degradation | - | 4 (5%) | 2 (3%) | 3 (4%) |
|  | Aromatic Compound Degradation | 2 (2%) | 9 (11%) | 2 (3%) | 10 (13%) |
|  | C1 Compound Utilization and Assimilation | 4 (5%) | - | 3 (4%) | - |
|  | Carbohydrate Degradation | 1 (1%) | 5 (6%) | 3 (4%) | 3 (4%) |
|  | Carboxylate Degradation | 1 (1%) | 5 (6%) | 2 (3%) | 2 (3%) |
|  | Fatty Acid and Lipid Degradation | - | 1 (1%) | 1 (1%) | - |
|  | Inorganic Nutrient Metabolism | 1 (1%) | 3 (4%) | 2 (3%) | 3 (4%) |
|  | Nucleoside and Nucleotide Degradation | 1 (1%) | 1 (1%) | 1 (1%) | - |
|  | Secondary Metabolite Degradation | - | 5 (6%) | - | 3 (4%) |
|  | Other | - | 1 (1%) | - | - |
| Detoxification | Antibiotic Resistance | - | 1 (1%) | - | 1 (1%) |
| Generation of Precursor Metabolite and Energy | Fermentation | 2 (2%) | 1 (1%) | 2 (3%) | - |
|  | Glycolysis | 1 (1%) | 2 (3%) | 2 (3%) | - |
|  | Glyoxylate cycle | - | - | - | 1 (1%) |
|  | Pentose Phosphate Pathways | 1 (1%) | 0 | 2 (3%) | - |
|  | TCA cycle | 4 (5%) | 1 (1%) | 2 (3%) | - |
| Macromolecule Modification | Nucleic Acid Processing | 1 (1%) | 1 (1%) | - | 1 (1%) |
| Superpathways | Superpathway of glyoxylate bypass and TCA | - | - | - | 1 (1%) |
|  | Superpathway of hexuronide and hexuronate degradation | - | 1 (1%) | - | - |
|  | Superpathway of histidine, purine, and pyrimidine biosynthesis | - | 1 (1%) | - | 1 (1%) |
| Total | | 84 (100%) | 80 (100%) | 70 (100%) | 78 (100%) |
